# Low levels of IgG2 and pneumococcal antibodies as predictors of benefit from IgG replacement in IgG subclass deficiency

**DOI:** 10.70962/jhi.20250188

**Published:** 2026-03-03

**Authors:** Per Wågström, Katarina Nyström, Janne Björkander, Mats Nilsson, Charlotte Dahle, Åsa Nilsdotter-Augustinsson, Lillemor Skattum, Sofia Nyström

**Affiliations:** 1 Department of Infectious Diseases, Ryhov County Hospital, Jönköping, Sweden; 2 Clinical Department of Infectious Diseases in Östergötland, Region Östergötland, Linköping, Sweden; 3 Department of Biomedical and Clinical Sciences, Linköping University, Linköping, Sweden; 4 Division of Clinical Immunology, Department of Clinical and Experimental Medicine, Faculty of Health Sciences, Linköping University, Linköping, Sweden; 5 Department of Health, Medicine and Caring Sciences, Linköping University, Linköping, Sweden; 6 Department of Clinical Immunology and Transfusion Medicine, Region Östergötland, Linköping, Sweden; 7 Division of Inflammation and Infection, Department of Biomedical and Clinical Sciences, Linköping University, Linköping, Sweden; 8 Department of Laboratory Medicine, Section of Microbiology, Immunology and Glycobiology, and Clinical Immunology and Transfusion Medicine, Skåne University Hospital, Lund University, Lund, Sweden

## Abstract

Immunoglobulin G subclass deficiencies (IgGSD) are associated with recurrent respiratory tract infections. Tools to identify patients with IgGSD who benefit from immunoglobulin G replacement therapy (IgGRT) are lacking. This crossover study evaluated the number of antibiotic-demanding infections on and after up to 18 mo off IgGRT in 28 patients with IgGSD. Pneumococcal antibodies against 21 serotypes were assessed using a multiplex assay. After 12 mo on IgGRT, the frequency of infections was reduced during the following 6 mo compared with the last 6 mo without IgGRT, indicating a delayed therapeutic effect. Low levels of pneumococcal serotype-specific antibodies and lower IgG2 levels were associated with the need to restart IgGRT. In conclusion, IgGRT effectively reduces bacterial infections in IgGSD, but the benefits may take at least a year to manifest. Pneumococcal antibody profiling and IgG2 levels may help identify patients needing long-term IgGRT.

## Teaser

Wågström et al. report that poor pneumococcal antibody protection and low IgG2 levels predict the need for continuous immunoglobulin therapy in IgG subclass deficiency. Their findings highlight the delayed benefit of IgRT and suggest pneumococcal antibody profiling as a tool for guiding long-term treatment decisions.

## Introduction

The role of immunoglobulin G replacement therapy (IgGRT) in IgG subclass deficiency (IgGSD) remains unclear, and international consensus on which patients should receive treatment is lacking. IgGSD is classified among inborn errors of immunity (IEI) with predominant antibody deficiencies (PAD). Although relatively common, IgGSD typically presents with a mild clinical phenotype, which may be aggravated by reduced levels of IgA (1). IgGSD is a heterogeneous disorder with variable infection susceptibility and inconsistent vaccine responses (2, 3, 4).

According to retrospective data, IgGRT appears to reduce the frequency of serious respiratory infections in selected patients with IgGSD (5), yet the criteria for identifying those who will benefit most from IgGRT remain unclear, and it has recently been proposed that given the cost and potential side effects of long-term IgGRT, prophylactic antibiotics may be effective in preventing infections in IgGSD (6). Additionally, it has been suggested that low-dose IgGRT may reduce infections in this group of patients and prevent the development of lung damage (7). Recurrent respiratory tract infections pose a risk for the development of bronchiectasis (8), and IgGSD is associated with exacerbations in individuals with chronic obstructive pulmonary disease (9). In patients with IgA deficiency, concomitant IgG2 and IgG3 subclass deficiencies are associated with reduced lung function (10). In Sweden, it has, since the late 1970s, been standard practice to consider IgGRT for patients with IgGSD complicated by recurrent bacterial infections, and the decision whether to introduce IgGRT or not is based on the frequency and severity of bacterial infections and/or the presence of structural lung damage (11).

Poor vaccine responses are hallmarks of PAD, and similarly, impaired IgG vaccine responses are observed in some patients with IgGSD (1). In IgGSD, poor responses to pneumococcal polysaccharide vaccines have been shown to correlate with low pre-immunization levels of serotype-specific IgG antibodies (SSA) (12, 13). In contrast to other PAD, poor responses to protein-conjugated polysaccharide vaccines, or the tetanus vaccine, are generally not observed in IgGSD (14, 15).

The levels of IgG subclasses vary significantly among healthy individuals, which limits their use as predictors of the need for IgGRT in IgGSD (16). Other components of the immune system may also contribute to the susceptibility to infections observed in IgGSD (17). For instance, polymorphisms in Fc-γ receptors and complement deficiencies may exacerbate infection risk in these patients through impaired antibody-mediated immune responses (18, 19, 20).

In the present study, we evaluated infection rates in 28 individuals with IgGSD during 18 mo of IgGRT and 18 mo without IgGRT. Specific pneumococcal antibody concentrations and complement function were assessed and correlated to infection rates and lung function. The overall aim was to assess the benefit from IgGRT in this population.

## Results

### Characteristics of the study population

**Figure 1.**
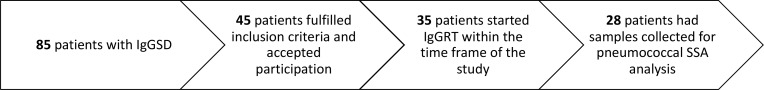
**Enrollment of study participants.** Of 85 patients with IgGSD, 45 met the inclusion criteria of the study: no known severe lung disease (FEV1% <40) and no previous discontinuation trial. 35 accepted to participate in the study and started IgGRT within the time frame of the study. Samples for analysis of pneumococcal SSA were available from 28 of the 35 patients who started IgGRT.

28 study participants with IgGSD (10 males and 18 females; median age 59 years, range 32–75) were included in the study (Table 1 and Fig. 1). 13 patients had received pneumococcal vaccination, mostly more than 7 years prior to sample collection. Chronic lung disease occurred in 43% and bronchiectasis in 29%. All participants completed an IgGRT discontinuation period of at least 6 mo. Among those who required continued IgGRT after the study, eight remained off treatment for 18 mo, seven for 10–17 mo, and two for at least 6 mo. Autoimmune diseases were frequent in the cohort, affecting 54% of patients (Table 1). Psoriasis and diabetes mellitus were observed in more than one individual. Two patients presented with multiple autoimmune conditions: one with autoimmune hemolytic anemia, Sjögren’s syndrome, and autoimmune thyroiditis; and another with diabetes mellitus, autoimmune thyroiditis, microscopic colitis, and celiac disease. Among baseline characteristics (Table 1), complex IgGSD (more tha one affected IgG subclass) was more common in participants who required IgGRT after the study, whereas repeated prednisone taper or continuous prednisone therapy was more common in participants who remained off IgGRT. All other characteristics were similar between participants who remained off IgGRT and those who required re-initiation or continuous IgGRT. However, there was a trend toward lower total IgG levels (P = 0.080), in the latter group.

**Table 1.** Demographics and clinical characteristics of study participants

Characteristic	Total (*n* = 28)	Cont IgGRT (*n* = 17)	No IgGRT (*n* = 11)	P value
Sex, male, *n* (%)	10 (36)	5 (29)	5 (45)	ns
Age, years, median (range)	59 (32–75)	58 (32–71)	60 (43–75)	ns
Years since diagnosis, median (range)	2 (0–30)	1 (0–30)	3 (1–21)	ns
scIgGRT, *n* (%)	27 (96)	16 (94)	11 (100)	ns
Dose IgGRT, mg/kg/wk, median (range)	103 (50–174)	101 (92–174)	103 (50–120)	ns
Ever vaccinated^a^, *n* (%)	13 (46)	8 (41)	5 (45)	ns
Infections with IgGRT, median (range)	2 (0–13)	2 (0–8)	2 (0–13)	ns
Infections without IgGRT, median (range)	3 (0–7)	3 (0–7)	2 (0–6)	ns
Months without IgGRT, median (range)	18 (6–19)	17 (6–18)	18 (18–19)	–
Chronic lung disease^b^, *n* (%)	12 (43)	7 (41)	5 (45)	ns
Bronchiectasis, *n* (%)	8 (29)	4 (24)	4 (36)	ns
Lung function %^c^, median (range)	91 (40–114)	90 (40–114)	91 (50–107)	ns
Autoimmunity, *n* (%)	15 (54)	10 (59)	5 (45)	ns
Systemic corticosteroids^d^	6 (21)	1 (6.0)	5 (45)	0.022
Immune suppression	0	0	0	–
Subnormal total IgG, *n* (%)	15 (54)	11 (65)	4 (36)	ns
Subnormal IgA, *n* (%)	6 (21)	5 (29)	1 (9.0)	ns
Subnormal IgG1	5 (18)	5 (29)	0	ns
Subnormal IgG2	11 (39)	9 (50)	2 (18)	ns
Subnormal IgG3	18 (64)	11 (65)	7 (64)	ns
Complex^e^ IgGSD, *n* (%)	9 (33)	8 (47)	1 (9.0)	0.047
MBL deficiency, *n* (%)	6 (21)	3 (18)	3 (27)	ns
IgM, g/L, median (range)	0.82 (0.07–3.0)	0.81 (0.07–2.3)	0.84 (0.3–3.0)	0.27–2.1
IgG, g/L, median (range)	6.2 (2.1–12)	5.4 (2.2–11.9)	8.2 (4.9–11.3)	6.7–15
IgA, g/L, median (range)	1.5 (0.09–3.8)	1.5 (0.09–2.8)	1.7 (0.84–3.8)	0.88–4.5
IgG1, g/L, median (range)	3.8 (1.4–10)	3.6 (1.4–10.3)	4.1 (3.3–9.0)	2.8–8.0
IgG2, g/L, median (range)	1.6 (0–4.5)	1.0 (0–4.5)	2.4 (0.55–4.2)	1.15–5.7
IgG3, g/L, median (range)	0.16 (0–1.0)	0.12 (0–0.9)	0.2 (0–0.5)	0.24–1.3

Cont, need for continued therapy after the study; MBL, mannose-binding lectin.

a Pneumococcal vaccination 1–20 years prior to blood sample collection.

b Asthma or chronic obstructive pulmonary disease.

c FEV1%.

d >1 prednisone taper or >5 mg daily prednisone.

e Deficient in >1 IgG subclass.

### IgGRT more than 12 mo reduced the burden of airway infections

**Figure 2.**
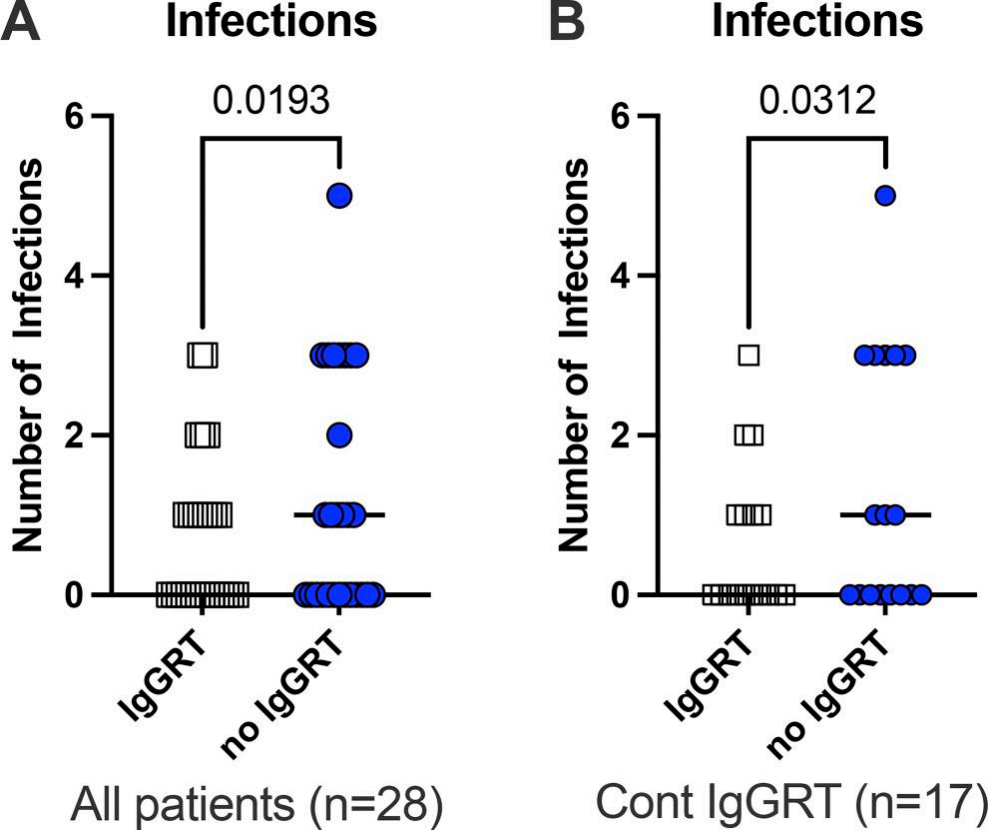
**IgGRT reduced the burden of airway infections in patients with IgGSD. (A)** Number of airway infections treated with antibiotics or infections that required hospitalization in the whole cohort of patients (*n* = 28) was reduced during months 12–18 on IgGRT compared with the last 6 mo without IgGRT **(B)** Number of infections treated with antibiotics during months 12–18 on IgGRT and during the last 6 mo without IgGRT in the subset of patients (*n* = 17) with the need for continuous (Cont) IgGRT. P values refer to the Wilcoxon test for paired analysis.

In the overall study population, the infection rates were similar when comparing the full 18-mo periods on and off IgGRT (Table 1). However, when focusing on the final 6 mo of each phase (months 12–18 when on IgGRT and the last 6 mo of the discontinuation trial, respectively), the rate of antibiotic-demanding respiratory tract or serious infections was lower during IgGRT across the entire cohort (Fig. 2 A). This reduction in infections remained significant in the subgroup that required continued IgGRT (Fig. 2 B) but was not observed in those who remained off treatment. These findings suggest that a reduced infection frequency after 12 mo of IgGRT is associated with a continued need for IgGRT.

### Complement function

No patient had a defective alternative or classical complement pathway, while the functional complement analysis detected deficiency in the mannose-binding lectin (MBL) pathway in six of the patients with IgGSD (Table 1). The presence of MBL deficiency was evenly distributed between those who needed to continue IgGRT after the study and those who did well without IgGRT.

### Prevalence of pneumococcal serotype-specific IgG antibodies (SSA) associates with the necessity of IgGRT

**Figure 3.**
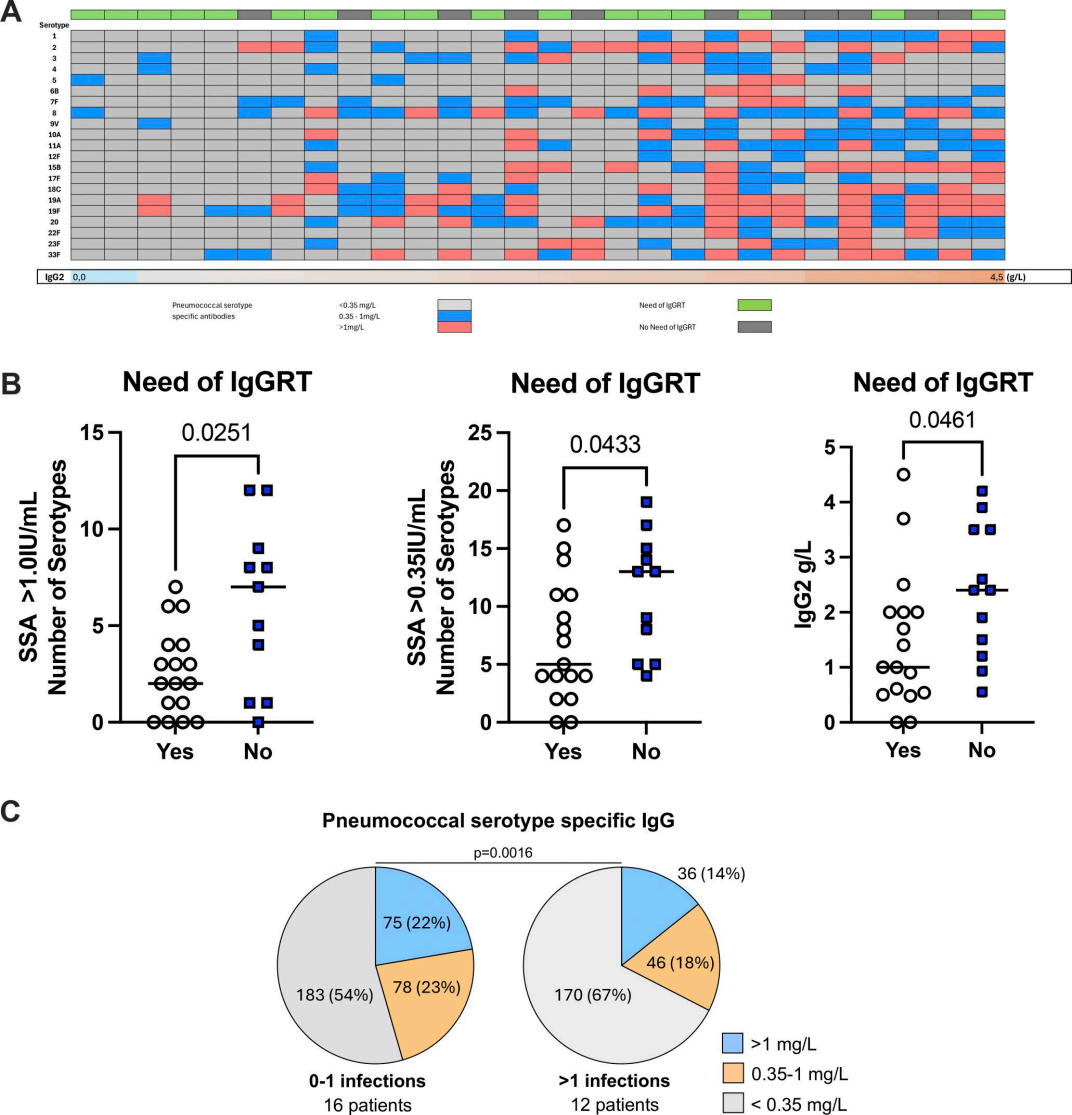
**Prevalence of pneumococcal SSA associates with the need for continuous IgGRT and infections. (A)** Prevalence of SSA in study participants and the association with IgG2 plasma levels. **(B)** Number of detected SSA and plasma levels of IgG2, in patients with the need for and without the need for continuous IgGRT after the discontinuation trial, respectively. **(C)** Percentages of SSA at protective levels (>0.35 mg/L) in groups of patients with low incidence or high incidence of infections (i.e., more than one airway infection treated with antibiotics or any infection requiring hospitalization) when off IgGRT. Mann–Whitney test or Fisher’s test was used for statistical analyses.

Only six patients had received pneumococcal vaccination within 5 years prior to sample collection for analysis of pneumococcal SSA. Analysis of pneumococcal SSA was performed on one occasion when the patients had been off IgGRT for at least 6 mo. Low levels, in this study defined as <0.35 mg/l, of pneumococcal SSA associated with low levels of IgG2 (Fig. 3 A). Among patients who needed continued IgGRT, fewer had protective levels (>0.35 mg/l) of pneumococcal SSA than the group who did well without IgGRT (Fig. 3 B). However, protective levels of pneumococcal antibodies to a restricted number of serotypes were found among patients with more infections (Fig. 3 C). Taken together, the prevalence of pneumococcal SSA associated with low levels of IgG2, the number of infections, and the need for continuous IgGRT.

### IgG2 levels associate with protective levels of pneumococcal SSA

**Figure 4.**
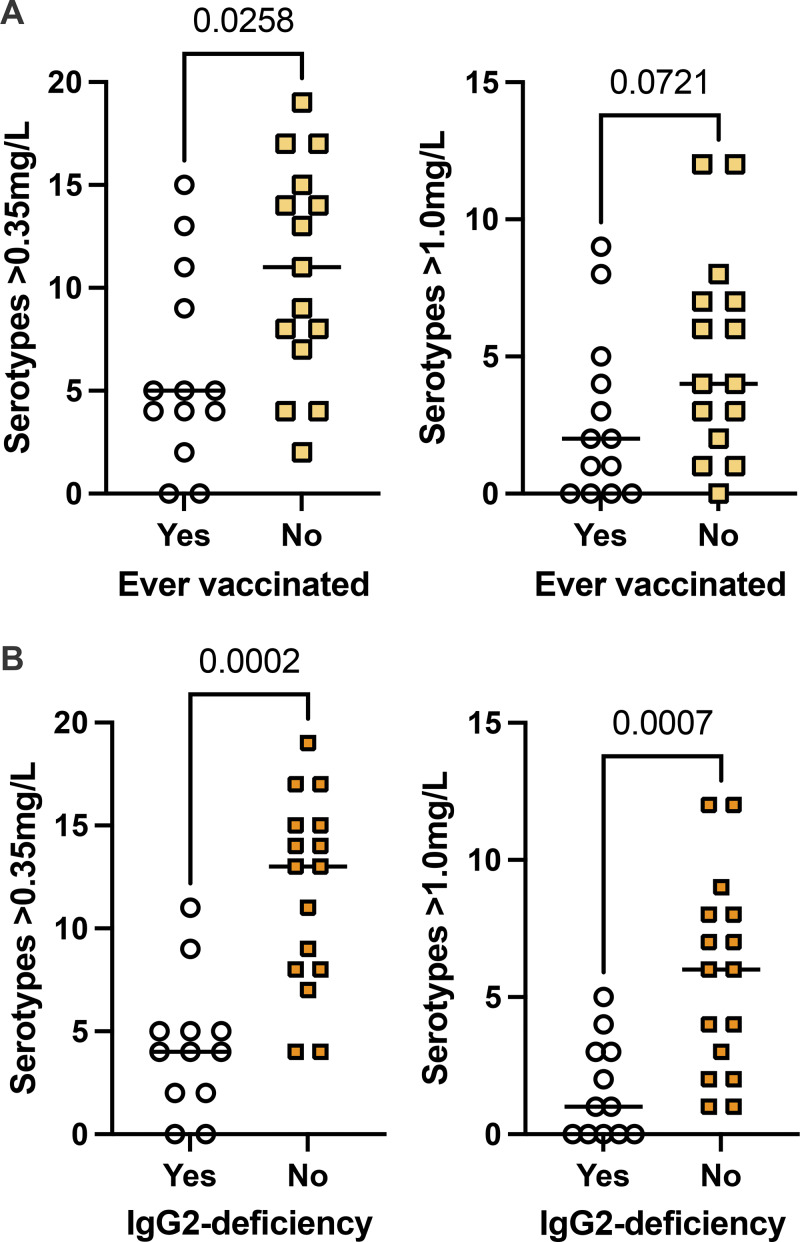
**IgG2 levels associate with protective levels of pneumococcal SSA. (A)** Patients who ever experienced pneumococcal vaccination had lower numbers of SSA at protective levels. **(B)** Patients with subnormal IgG2 had lower numbers of pneumococcal SSA at protective levels. Mann–Whitney test was used for statistical analyses.

Patients who ever had received pneumococcal vaccination had protective levels of antibodies to lower numbers of pneumococcal serotypes than those who were unvaccinated (Fig. 4 A). Among ever vaccinated patients, subnormal IgG2 was more common, affecting 9 of 13 patients (Fig. 4 B). In summary, subnormal IgG2 concurred with increased infection rates, and low levels of pneumococcal SSA.

### Correlation analysis and predictors of IgGRT need

**Figure 5.**
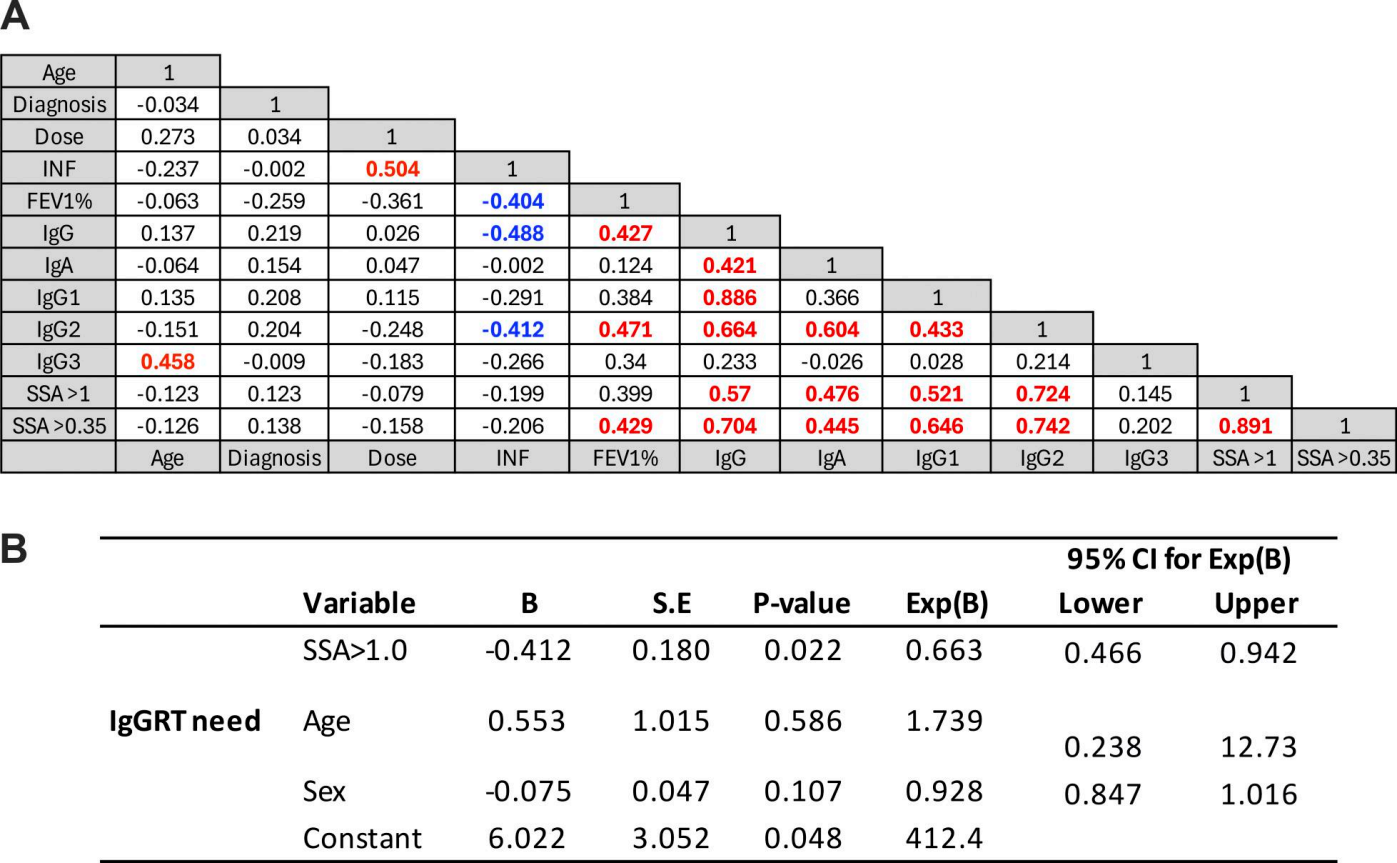
**Correlations of clinical and immunological parameters and predictors of continued IgGRT need.** The matrix illustrates pairwise correlations among continuous variables in the study cohort (*n* = 28). Strong positive correlations (Spearman) were observed between total IgG and IgA, IgG1, IgG2, and pneumococcal SSA response counts above 1 mg/ml, while IgG3 showed no significant correlation with these parameters. FEV1% correlated moderately with total IgG, IgG2, and SSA counts. **(A)** Positive correlation >0.4 is indicated by red color, and negative correlation <−0.4 by blue color. **(B)** Logistic regression analysis identified SSA >1.0 mg/ml as the only significant predictor of IgGRT need, also when adjusting for age and sex yielded similar results. Diagnosis indicates years since diagnosis. Dose, weekly dose of IgGRT; INF, number of infections during the last 6 mo without IgGRT; SSA>1 and SSA>0.35, pneumococcal SSA count >1.0 mg/ml and 0.35 mg/ml, respectively. B, unstandardized regression coefficient; SE, standard error.

To explore the relationships between the variables, a correlation matrix was constructed using Spearman’s rank correlation coefficient (Fig. 5 A). The matrix revealed several significant associations, indicating potential interdependencies among the measured parameters. Total IgG correlated with IgA, IgG1, and IgG2, as well as with pneumococcal SSA, but not with IgG3. Lung function measured as FEV1% ranged between 40 and 114% in the cohort, and six patients had impaired lung function. FEV1% correlated moderately (r_s_ > 0.4) with total IgG, IgG2, and pneumococcal SSA counts, whereas IgG3 levels did not correlate with FEV1% (Fig. 5 A). The number of infections correlated with dose IgGRT and showed an inverse correlation with FEV1% and IgG. A moderate correlation between IgG2 and IgG1 plasma levels suggests that subnormal IgG1 may exacerbate lung damage in patients with low levels of IgG2. To identify predictors of IgGRT need, a backward stepwise logistic regression was performed including complex IgGSD (more than one affected IgG subclass), number of pneumococcal SSA >1.0 mg/ml, age, and sex. Variables with P >0.2 were sequentially removed. The final model retained SSA >1.0 mg/ml as the only significant predictor, indicating that a higher number of protective SSA responses were associated with a lower likelihood of requiring IgGRT (odds ratio 0.714, 95% confidence interval 0.536–0.952, P = 0.022). As a sensitivity analysis, the variable number of pneumococcal SSA >1.0 mg/ml was also found to predict the need for continued IgGRT in a model adjusted for age and sex, yielding similar results (Fig. 5 B).

## Discussion

We found that subnormal IgG2 was associated with low levels of pneumococcal SSA despite vaccination, reduced FEV1%, and a continued need for IgGRT. The age and sex distribution of subjects in the present cohort was similar to those in other studies investigating IgGSD and IgGRT (6, 7). The frequency of chronic lung disease and bronchiectasis was lower than that of other cohorts (7), although 17 out of 28 participants (60%) needed to restart IgGRT due to increased infection rates. The decrease in infection rate became statistically significant after 12 mo of IgGRT, when the levels of IgG and IgG subclass were normalized.

The delayed decrease in infection rate may reflect a gradual recovery of the immune system as a result of sustained normal IgG levels. This finding is in contrast to a previous study in patients with IgGSD and asthma, where the initiation of IgGRT reduced infection rates and improved asthma control already after 6 mo of treatment in IgGSD (21). We have previously observed that IgGRT improved B cell activation and increased the number of regulatory T cells, changes that may also play a role in infection control (19). Together, our results support a gradual immune recovery during IgGRT, which may also explain the positive outcomes seen with low-dose IgGRT in IgGSD (7).

As a group, patients who required continued IgGRT after the study had fewer protective pneumococcal SSA across the 21 tested serotypes, which may explain why they were more likely to benefit from IgGRT. This finding supports the utility of extended pneumococcal SSA profiling in the evaluation of patients with IgGSD (22, 23). Notably, patients who had ever received pneumococcal vaccination exhibited lower levels of pneumococcal SSA, presumably reflecting the presence of increased respiratory tract infections that led to their initial vaccination. Patients with low IgG2 have an increased rate of lower respiratory tract infections with encapsuled bacteria than patients deficient in IgG1 or IgG3 (3). Poorer serological protection in patients with subnormal IgG2 reflects that responses against pneumococcal capsular polysaccharide antigens are predominantly restricted to IgG2 (24, 25).

The group of patients with a higher burden of antibiotic-demanding infections were characterized by a lower prevalence of protective levels of pneumococcal SSA. The conjugate pneumococcal vaccine has been shown to elicit protective immunity and increased opsonophagocytic activity in patients with IgGSD and may be of clinical benefit in patients with IgGSD (26, 27). This is particularly relevant for patients with subnormal IgG2 and impaired antibody responses to polysaccharide antigens, where further studies on the efficacy of conjugate vaccines are warranted, especially given the association with lung damage (28).

The study revealed interdependencies among several immunological variables, including total IgG and IgA levels, IgG1 and IgG2 subclasses, and pneumococcal SSA responses. In addition, lung function correlated with immunological parameters and was inversely related to the frequency of infections, highlighting the link between humoral immunity and pulmonary integrity. However, no associations were observed between age or years since diagnosis and the other measured variables. The observed correlations between FEV1% and IgG1 levels in the cohort suggest that other pathogens, beyond encapsulated bacteria, may also play a role in lung function decline. Growing evidence points to a contribution from viral pathogens in infection-related lung damage (29). We have previously shown that patients with IgGSD exhibit a state of low-grade systemic inflammation, which may be sustained by unresolved or recurrent respiratory tract infections (30, 31). Together, these findings illustrate the vulnerability of patients with IgGSD and poor pneumococcal SSA protection.

MBL deficiency, affecting 5–20% Caucasians, is the most common complement deficiency (32). In our cohort, 21% of the patients had concurrent MBL deficiency, consistent with previous findings in both IgGSD and healthy European populations (33). In the present study, patients with MBL deficiency were evenly distributed between those who required continued IgGRT and those who did not. To determine whether MBL deficiency contributes to increased infection susceptibility in patients with IgGSD, significantly larger studies are needed.

The etiology of IgGSD is poorly understood, and multiple mechanisms likely contribute to low IgG subclass levels. The complexity of the disease is reflected in the heterogeneity of the clinical presentations in this cohort. Subnormal IgG or subclass levels are common findings in monogenic IEI (34). The relatively high prevalence of autoimmune manifestations of this cohort, including two cases of multiple autoimmunity, suggests that patients with monogenic conditions might have been included. Furthermore, it was recently reported that several monogenic IEI can present with subnormal IgG2 levels and impaired response to polysaccharide antigens (15). In other countries, the subgroup of patients with subnormal IgG would typically be classified as having unclassified antibody deficiency instead of IgGSD. Unclassified antibody deficiency represents a broader and even more heterogeneous PAD than IgGSD (35). Both conditions, however, share diagnostic uncertainty and may include individuals with undetected monogenic IEI, whose immunodeficiency could progress over time to a more severe phenotype. The absence of genetic testing limits the generalizability of our findings. Additional limitations of this study include the small cohort size and the retrospective evaluation vaccination and vaccination responses. However, the reliability of the findings is strengthened by the study design, where each participant serves as their own control when evaluating infection rates with and without IgGRT, as well as by the long follow-up period.

In conclusion, this crossover study showed that poor pneumococcal antibody protection associated with low IgG2 and predicted the need for continuous immunoglobulin therapy in patients with IgGSD. The reduction of antibiotic-demanding infections was evident only after 12 mo on IgGRT.

## Materials and methods

### Ethics

The study protocol was approved by the Regional Ethical Review Board in Linköping, Sweden (Dnr. 2011/506-31). Written informed consent was obtained from all study participants.

### Definition of IgGSD

This prospective study was conducted at two Swedish regional centers of infectious disease during 2012–2015. According to the Swedish classification, IgGSD is defined by reduced levels of IgG1, IgG2, and/or IgG3 and may also be associated with subnormal levels of IgA or moderately decreased total IgG (11). Serum IgG4 levels are not considered in the Swedish criteria for IgGSD, since IgG4 is of limited importance in antimicrobial defense (25). In addition to reduced levels of one or more IgG subclasses, a history of recurrent bacterial infections (at least four bacterial infections per year, over a period of 2 years) is needed to fulfill the diagnostic criteria of IgGSD. Reduced IgG subclass levels were defined as plasma concentrations below the reference range: IgG1 <2.8 g/L, IgG2 <1.15 g/L, and IgG3 <0.24 g/L (16).

### Study population and design of the study

Out of 85 adult patients diagnosed with IgGSD, 35 (22 women and 13 men) fulfilled the inclusion criteria: no severe lung disease and no previous IgGRT discontinuation trial. Severe lung disease was defined as forced expiratory volume in 1 s (FEV1) <40% of the predicted value, recognizing that interruption of IgGRT may worsen pulmonary function in this particularly susceptible subgroup. The cohort, which has previously been described (19, 31), has not been subjected to any genetic testing or evaluation of vaccine responses. None of the study participants received prophylactic antibiotics before or during the study. Participants received IgGRT for a period of 18 mo, followed by a discontinuation phase lasting between 7 and 18 mo. One participant received intravenous IgGRT, while the others received subcutaneous administration. The median dose was 103 mg/kg/wk (range 50–174 mg/kg/wk). The frequency of infections requiring antibiotic treatment was assessed during both treatment and discontinuation phases and served as the basis for determining the need for continued IgGRT. In six cases, IgGRT was reinitiated prior to the end of the 18-mo discontinuation period due to a high burden of antibiotic-demanding infections, i.e., two verified bacterial respiratory tract infections or one serious infection. According to the study protocol, a minimum of 6 mo without IgGRT was required, to avoid interference from residual immunoglobulin from IgGRT in laboratory analyses. Samples collected during the off-treatment phase were available from 28 participants (18 women and 10 men), who comprise the cohort of this study (Fig. 1). Information about lung function, FEV1%, was retrieved from patients’ medical records, as well as information about any previous pneumococcal vaccination.

### Handling of blood samples

Blood samples were drawn in vacutainers. Levels of IgG, IgA, IgM, and IgG subclasses IgG1, IgG2, and IgG3 were measured according to standard operating procedures at the Laboratories of Clinical Chemistry in Region Jönköping’s County and Region Östergötland before the initiation of IgGRT. Sera/plasmas were separated within 6 h of blood collection and were stored at −80°C until profiling of pneumococcal SSA and complement function testing.

### Complement function test

Function of the classical, alternative, and MBL complement activation pathways was evaluated in serum by using the semi-quantitative enzyme-linked immunosorbent assays (ELISAs) WIESLAB Complement System Classical Pathway (COMPLCP310), WIESLAB Alternative Pathway (COMPLAP330), and WIESLAB MBL Pathway (COMPLMP320) from SVAR Life Science. The ELISAs were used according to the manufacturer’s instruction. In brief, the wells were coated with specific activators of the respective pathway, and the level of activation was assessed by detection of C9 in the membrane attack complex.

### Detection of serotype-specific pneumococcal antibodies

Pneumococcal serotype-specific IgG concentrations were determined for 21 capsular serotypes (1, 2, 3, 4, 5, 6B, 7F, 8, 9V, 10A, 11A, 12F, 15B, 17F, 18C, 19A, 19F, 20, 22F, 23F, and 33F), using a newly developed in-house multiplex fluorescent magnetic microsphere immunoassay. The method is mainly based on the procedure described by Lal et al. (36) with some modifications (37). Reference standard serum 007sp, which has assigned IgG antibody concentrations for the included serotypes, was used for calibration and expression of results in mg/l (38, 39, 40). A threshold of 0.35 mg/L was used to define protective levels of pneumococcal serotype-specific IgG, as this concentration is associated with clinical protection against invasive pneumococcal disease (27, 41).

### Logistic regression analyses

To explore factors associated with the need for IgGRT, we performed logistic regression analyses with continued need for IgGRT as the dependent variable. Independent variables initially considered were age, sex, complex IgGSD, and the number of pneumococcal SSA >1.0 mg/ml. Due to the limited sample size and risk of overfitting, we applied a backward stepwise selection procedure to identify the most relevant predictors. Variables with P >0.2 were sequentially removed. In addition, as a sensitivity analysis, we constructed a simplified model including SSA >1.0 mg/ml, adjusted for age and sex.

### Statistical analyses

For comparisons between two groups, the Mann–Whitney U test was used, the Wilcoxon test for paired samples, the Spearman test for correlation analyses, and Fisher’s test for categorical data. Statistical significance was defined as P < 0.05. IBM Statistical Package for the Social Sciences version 29.0.2.0 was used for regression analyses, and GraphPad Prism 9.3.1 (GraphPad Software) was used for other calculations and graphics.

## Ethics approval

This study was performed in line with the principles of the Declaration of Helsinki. Approval was granted by the Ethics Committee of Linköping University (Dnr. 2011/506-31). Written informed consent was obtained from all participants included in the study.

## Data Availability

The datasets generated during and/or analyzed during the current study are available from the corresponding author on reasonable request.
